# Do information publicity and moral norms trigger waste-sorting intention among households? A sequential mediation analysis

**DOI:** 10.3389/fpsyg.2023.1193411

**Published:** 2023-06-15

**Authors:** Yan Li, Muhammad Yaseen Bhutto, Chaojing Sun, Syed Muntazir Mehdi

**Affiliations:** ^1^Business School, Zhengzhou University, Zhengzhou, China; ^2^Postdoctoral Mobile Research Station of School of Politics and Public Management, Zhengzhou University, Zhengzhou, China; ^3^Business School, Shandong Jianzhu University, Jinan, China; ^4^Shandong Labor Vocational and Technical College, Jinan, China; ^5^Institute of Health and Business Management, Jinnah Sindh Medical University, Karachi, Pakistan

**Keywords:** information publicity, moral norms, green norms, perceived behavioral control, attitude, subjective norms, waste sorting intention

## Abstract

**Introduction:**

The quick pace of technological advancement and urbanization has led to a significant increase in waste production, severely damaging environmental quality and human health. The sorting of waste is a viable option to reduce environmental hazards and attain high recovery rates in the cities. This research extended the theory of planned behavior (TPB) by integrating information publicity (IP) and moral norms (MNs).

**Methods:**

A conceptual model has been developed to explore the predictors of waste-sorting intention of households. The data from 361 Pakistani households have been collected using the purposive sampling method and analyzed via PLS-SEM.

**Results and discussion:**

The study's results revealed that IP is important in creating awareness and establishing moral norms regarding waste sorting among households. The findings further confirm that MN, attitude (ATD), subjective norms (SNs), and perceived behavioral control (PBC) sequentially mediate between IP and WSI. The findings of the current study provides useful practical implications to the practitioners and academicians to combat environmental pollution.

## Introduction

Every year, the world produces at least 1.47 billion tons of municipal solid waste (Shen et al., 2022). The World Bank (2019) reported that solid waste production globally reached ~2 billion tons in 2016, and it is expected to increase to 3.40 billion tons by the year 2050, primarily due to substantial population growth and the rapid advancement of industrialization. Maintaining such large quantities of solid waste requires a significant financial investment. The issue of waste is becoming a growing concern from social, economic, and environmental perspectives, both in developed and developing nations. In past, many researchers have indicated the significance of waste reduction towards the accomplishment of financial, social, and fair and healthy system (Kim et al., 2019; Amicarelli et al., 2020).

According to estimates, from agricultural production to final consumption generates over 1.3 billion tons of food waste, leading to environmental consequences of ~3.3 gigatons of CO_2_ equivalent each year (Amicarelli et al., 2021). From a worldwide perspective encompassing environmental, financial, and social aspects, food waste was estimated at ~US $1 trillion annually. This estimate includes environmental costs amounting to US $700 billion and social costs amounting to US $900 billion. The average estimates depict a loss of ~$680 billion to developed countries and less than $310 billion to developing countries (United Nations Environment Programme, 2021). In the early 1990's, Asian nations spent over US $25 billion annually on solid waste management; by 2025, this amount is predicted to reach ~US $50 billion (Al-Maaded et al., 2012). Similarly, the volume of domestic garbage created in Pakistani cities has grown yearly due to urbanization (Korai et al., 2017). The country generates ~32.6 million metric tons of household waste annually at an average waste production of 0.43 kg per capita per day (Permana et al., 2015). In addition, the concerned authorities in the country still need to take the required steps to sort and manage the waste. However, waste burning and open dumping are common practices of garbage disposal in the country that cause severe air pollution and harm global warming (Iqbal et al., 2022). Waste sorting is essential to reduce environmental pollution because it allows for the proper disposal and recycling of materials. Unsorted waste is often dumped in landfills, which might take hundreds of years to degrade. As a result, it releases dangerous chemicals and pollutants into the environment.

Over the last few years, global waste management has become a significant cause of concern for policymakers and poses a threat to the ecosystem (Amicarelli et al., 2022; Aslam et al., 2022). As a result, wastes generated from household activities have severely affected humans and wildlife, such as damaging plants and increasing the risk of animal injury (Talpur et al., 2018). Furthermore, harmful chemicals are released into the air and soil when wastes are burned or buried in the ground, contaminating the environment (Ilyas et al., 2018). Therefore, a robust waste management strategy is essential in this dire circumstance, which may be more effectively accomplished using waste sorting. Waste sorting allows for materials that can be recycled or composted to be processed accordingly, reducing the amount of waste sent to landfills and improving environmental health. In the past, researchers have concentrated their efforts on recycling waste goods to achieve appropriate waste management in different countries (Dixit and Badgaiyan, 2016; Khan et al., 2019; Kumar et al., 2019).

Household waste significantly impacts environmental pollution (Moshood et al., 2022; Wang et al., 2022). The average household generates a significant amount of waste each day, including food scraps, packaging materials, and other items that are not biodegradable. These materials can end up in landfills or the ocean, harming wildlife and degrading natural habitats (Li et al., 2020; Shen et al., 2020). Households must recycle, reduce single-use items, and support efforts to reduce waste pollution (Vanapalli et al., 2021). Households can recycle waste by separating it into different types, such as bottles and containers, and putting them in a recycling bin. In addition, households can use reusable containers, bags, and water bottles to reduce waste (Jacobsen et al., 2018). By adopting sustainable behavior, households can significantly reduce the amount of waste that ends up in landfills and oceans, which in turn contributes to environmental pollution (Hameed et al., 2022). Waste sorting is an ethical and responsible action demonstrating a commitment to sustainability and future generations' wellbeing (Iqbal et al., 2022; Sharma et al., 2022). In this regard, information publicity (IP) and moral norms (MN) can be crucial in encouraging individuals to recycle plastic waste. Individual attitudes and behaviors toward the responsible disposal of plastic waste are influenced by MN (Rowlands et al., 2003; Ozaki, 2011). IP is an effective tool for promoting pro-environmental behavior by raising awareness, providing information, and building social support for sustainable actions (Meng et al., 2019; Waris et al., 2022). The belief that individual is responsible for protecting the environment, the significance of reducing waste, and using recycled materials is a more sustainable practice that reflects MN (Wan et al., 2021; Soomro et al., 2022). Individual values and priorities can significantly impact a person's willingness to reduce waste (Mintz et al., 2019; Sohoo et al., 2022). By conforming to these moral standards, people will be more inclined to waste sorting than dumping it irresponsibly. This can reduce waste pollution and the amount of waste in landfills and the environment.

Previous studies mainly focused on the direct effects of the TPB constructs on individual intention to waste recycling. However, they neglected the crucial roles of IP and MN related to households' waste-sorting intentions. Integrating IP and MN into TPB would help to understand households' inclination toward waste-sorting intention. Therefore, the current study will fill this gap by analyzing the indirect impact of information publicity on waste-sorting intention. In addition, the study will analyze the sequential indirect relationship between IP and WSI through moral norms and TPB constructs (ATD, SN, and PBC). Environmental management literature proves that TPB constructs significantly influence behavioral intention (Khan et al., 2019; Aboelmaged, 2021; Hameed et al., 2022). Therefore, understanding the significance of the factors via a novel framework is essential to mitigate the adverse impact of environmental pollution. Finally, the study findings would highlight the importance of IP in encouraging individuals to engage in waste-sorting behavior to improve environmental health.

In light of this, this study aims to understand household WSI in the context of developing countries. The study intends to explore the novel relationships between IP, MN, and TPB constructs (ATD, SN, and PBC) leading to WSI. Furthermore, it aims to understand the influence of motivating factors and novel nexus between the constructs leading to WSI. The findings of the study help policymakers and governments to design and implement strategies that motivate the household toward proper waste sorting.

## Literature review and theoretical background

### Theoretical framework: an extended theory of planned behavior (TPB)

The literature on environmental management depicts that several theories have been used to understand individual behavioral intentions. The theory of planned behavior (TPB) developed by Ajzen (1991) posits that an individual's intention to engage in a behavior is determined by their attitude toward that behavior, the perceived social norms related to that behavior, and the individual's perceived behavioral control. The norm activation model (NAM) posits that personal and social norms influence an individual's behavior (Onwezen et al., 2013; Goh et al., 2022). Recently, many researchers have used NAM to evaluate individual recycling intentions (Arkorful et al., 2022; Nketiah et al., 2022; Zhang et al., 2022). In addition, the value-belief-norm (VBN) model also explains and predicts recycling intention. According to the VBN model, an individual's behavior is influenced by their values (personal beliefs about what is essential), beliefs, and norms (Stern et al., 1999). VBN posits the belief that recycling effectively reduces waste and preserves resources (Whitmarsh et al., 2018) and the presence of social norms that support recycling (Stern et al., 1999). Previous studies indicate the significance of norms shaping individual pro-environmental behaviors. This study applied Ajzen's (1991) TPB to understand household behavior. The environmental management studies depict that AT, SN, and PBC have a significant role in understanding behavioral intentions (Arvola et al., 2008; Aboelmaged, 2021; Waris et al., 2021). Researchers found that TPB constructs significantly influence individual intention in the context of waste recycling (Khan et al., 2019; Aboelmaged, 2021; Hameed et al., 2022). Therefore, this study extends TPB theory by incorporating IP and MN as additional constructs to evaluate household waste-sorting intention.

### Hypothesis development

#### Information Publicity (IP)

Information Publicity is disseminating environmental problems and actions the public may do to promote environmental sustainability (Waris et al., 2022). This can include information about recycling programs, conservation efforts, and the impact of individual actions on the environment (Wang et al., 2018). Previous studies have shown that information publicity can effectively promote pro-environmental behavior (Wang et al., 2014). The study by Wang et al. (2018) on the effects of information publicity on residents' electronic waste found that individual norms favorably influence behavioral intentions. Other researchers also demonstrated that information dissemination publicity could significantly influence electronic waste recycling intentions (Chen and Chang, 2013). Steg (2008) posits that information publicity is crucial in successfully conserving energy. Wang et al. (2014) studied the predictors of energy conservation. They confirmed the positive impact of publicizing information on residents' attitudes and behavioral intentions. Researchers found that publicity information positively impacts individual norms and ATD toward electronic waste recycling but does not significantly affect PBC (Wang et al., 2018). However, Waris et al. (2022) study revealed the significant impact of information publicity on attitude and PBC. Previous studies have shown that publicity information significantly influences people's pro-environmental intentions (Wang et al., 2014, 2018; Waris et al., 2022). Based on previous studies findings, it can be assumed that information publicity has a significant role in household waste-sorting intention. Hence, we propose the following hypotheses:

*H*_1*a*_*: IP has a positive influence on WSI*.
*H*_1*b*_*: IP has a positive influence on MN*.
*H*_1*c*_*: IP has a positive influence on ATD*.
*H*_1*d*_*: IP has a positive influence on SN*.
*H*_1*e*_*: IP has a positive influence on PBC*.

#### Moral Norms (MNs)

Moral Norms establish an individual's ethical principles and opinions regarding their behavior (Beck and Ajzen, 1991; Armitage and Conner, 2001). MN is an essential factor that affects households' pro-environmental behavior (Rezvani et al., 2017; Lu et al., 2020). The TPB model does not reach conclusions about moral issues since it presupposes that behaviors result from volitional processing. However, when anticipating behaviors that have moral consequences, this exclusion is highly problematic (Conner and Armitage, 1998; Liu et al., 2020). Ajzen (1991) also argued that, in some cases, moral obligations toward a given behavior should be evaluated along with original TPB constructs. Conner and Armitage (1998) claimed that moral norms should similarly influence behavior involving moral obligations. Many researchers have included MN in the TPB and enhanced the explanatory power of the model (Yadav and Pathak, 2017; Meng et al., 2022). Tonglet et al. (2004) explored the predictors of recycling behavior and found that moral norms significantly impact intentions. The study by Onwezen et al. (2013) revealed that moral norms have a huge role in decision-making regarding pro-environmental behavior. Chan and Bishop (2013) study on household waste sorting and recycling indicated the significance of moral norms leading to pro-environmental behavior. Several recent studies confirmed the positive effects of MN on PBC, AT, and SN (López-Mosquera et al., 2014; Shin and Hancer, 2016; Tan et al., 2017). Based on the earlier findings, MN was found to be an essential factor in people's pro-environmental behavioral intention, and it positively impacts ATD, SN, and PBC. Thus, the following hypotheses are proposed:

*H*_2*a*_*: MN has a positive influence on WSI*.
*H*_2*b*_*: MN has a positive influence on ATD*.
*H*_2*c*_*: MN has a positive influence on SN*.
*H*_2*d*_*: MN has a positive influence on PBC*.

### The mediating effect of MN

Previous studies have found that moral norms significantly predict pro-environmental behavior (Chan and Bishop, 2013; Botetzagias et al., 2015). In the context of pro-environmental behavior, a meta-analysis by Klöckner (2013) showed that MN mediated the relationship between ATD and intention. In relation to this, Liu et al. (2020) conducted a study on Chinese consumer green purchases that revealed MN mediated the relationship between ATD and intention. The mediating effects of MN on the relationships between IP and intention have not been verified in previous studies. Many studies have confirmed the direct impact of MN on recycling intentions (Khan et al., 2019; Soomro et al., 2022). For example, researchers found direct effects of MN on intention (Chen and Tung, 2014). However, studies have not examined the relationship between IP, MN, and intention. Accordingly, this research seeks to find whether moral norms mediate the relationship between information publicity and intention. Hence, we propose the following hypothesis:

*H*_3_*: MN will positively mediate the relationship between IP and WSI*.

#### Attitude (ATD)

Attitude is a person's opinion and judgment of certain conduct (Fishbein et al., 1980). An individual demonstrates a positive ATD toward an activity if the individual expects a favorable outcome of their actions (Khan et al., 2019; Soomro et al., 2022). Recently, several studies examined the role of AT on an individual's BI (Khan et al., 2019; Hameed et al., 2022; Najmi et al., 2022). The positive effects of individuals' AT on their conduct have been reported in studies (Botetzagias et al., 2015; Wang et al., 2016). However, only a few studies empirically confirmed a positive link between ATD and behavioral intention (Dixit and Badgaiyan, 2016). Based on the previous evidence, it is believed that a person's ATD will positively influence SWI. Therefore, the following hypothesis is proposed:

*H*_4_*: ATD has a positive influence on WSI*.

#### Subjective norms (SNs)

The subjective norms refer to an individual's social pressure to engage in or refrain from a specific action or group of behaviors (Ajzen, 1991). A person's immediate surroundings, including family, friends, and others, create that pressure. Generally, it can be defined as the pressure society exerts on a person to act or perform a behavior according to society's expectations (Soomro et al., 2022). Several studies have shown that there is a positive impact of SN on behavioral intention (Chen and Tung, 2010; Echegaray and Hansstein, 2017; Waris et al., 2022). Therefore, SN is an essential factor that impacts behavioral intention. Based on the earlier results, it is believed that SN will positively impact SWI. Thus, the study proposes the following hypothesis:

*H*_5_*: SN has a positive influence on WSI*.

#### Perceived behavioral control (PBC)

Perceived behavioral control is the degree to which a person believes they have control over their behavior. The two main factors linked to behavioral performance are self-efficacy and perceived controllability (Ajzen, 1991). First, as an individual engages in an activity, the ease and difficulty with which the conduct is linked have an impact on their decision-making process. Second, a person's conduct may be influenced by how much control they have over the performance of a behavior. People are more engaged in behaviors they think are easy to achieve than those they think are hard and out of their control (Bamberg and Möser, 2007). Several studies have identified PBC as a crucial driver of individuals' behavioral intentions (Hameed et al., 2019; Liu et al., 2020). In the context of pro-environmental behavior, researchers found that PBC is a significant factor in plastic waste recycling (Hameed et al., 2022), recycling solid wastes (Soomro et al., 2022), adoption of solar energy (Waris et al., 2022), and eco-conscious behavior (Hameed et al., 2019). Based on the previous study evidence, it is believed that PBC will significantly influence SWI. Therefore, we propose the following hypothesis:

*H*_6_*: PBC has a positive influence on WSI*.

### Mediating effects of TBP constructs

Ajzen (1991) indicated that ATD, SN, and PBC are the three main predictors of behavioral intention. Researchers found that ATD, SN, and PBC are significant mediators in the TPB framework (Tarkiainen and Sundqvist, 2005; Kumar et al., 2017; Bourdage et al., 2020). Previous studies indicate that information publicity has a direct and indirect impact via TPB constructs on solid wastes (Soomro et al., 2022), electronic waste recycling intention (Wang et al., 2018), and adoption of solar energy (Waris et al., 2022). Furthermore, studies revealed that MN determines intention and is an important predictor of AT, SN, and PBC (Arvola et al., 2008; López-Mosquera et al., 2014; Soomro et al., 2022). Many recent studies provided empirical evidence of significant indirect effects of MN on intentions via AT, SN, and PBC (Klöckner, 2013; López-Mosquera et al., 2014; Wang et al., 2016; Liu et al., 2020). The literature provides enough evidence on the indirect effect of the MN on behavioral intentions via ATD, SN, and PBC. Thus, it is believed that MN indirectly impacts an individual's WSI. Therefore, the following hypotheses are proposed:

*H*_7*a*_*: AT will positively mediate the relationship between MN and WSI*.
*H*_7*b*_*: SN will positively mediate the relationship between MN and WSI*.
*H*_7*c*_*: PBC will positively mediate the relationship between MN and WSI*.

### Serial mediating effects of MN and TPB constructs

This study proposes a sequential indirect relationship between IP and WSI through MN and TPB constructs (ATD, SN, and PBC) based on the assumption that MN may not directly influence household WSI. It holds that MN significantly influences WSI (Khan et al., 2019; Wang et al., 2020). However, increasing the WSI household must have positive ATD, feel societal pressure, and accept that they can easily perform waste sorting. Wang et al. (2018) findings confirmed that ATD, SN, and PBC are essential mediating factors between IP and intention that signifies the importance of the IP leading to behavioral intention. Other researchers found significant direct and indirect impacts of moral norms and TPB constructs in the context of pro-environmental behavior (Khan et al., 2019; Liu et al., 2020; Soomro et al., 2022). Previous researchers indicated that IP and MN significantly improve behavioral intention. Accordingly, the current study posits that the relationship between IP and household WSI enhances via MN and TPB constructs. Therefore, we propose the following hypotheses:

*H*_8*a*_*: MN and ATD will serially mediate the relationship between IP and WSI*.
*H*_8*b*_*: MN and SN will serially mediate the relationship between IP and WSI*.
*H*_8*c*_*: MN and PBC will serially mediate the relationship between IP and WSI*.

**Figure 1:**
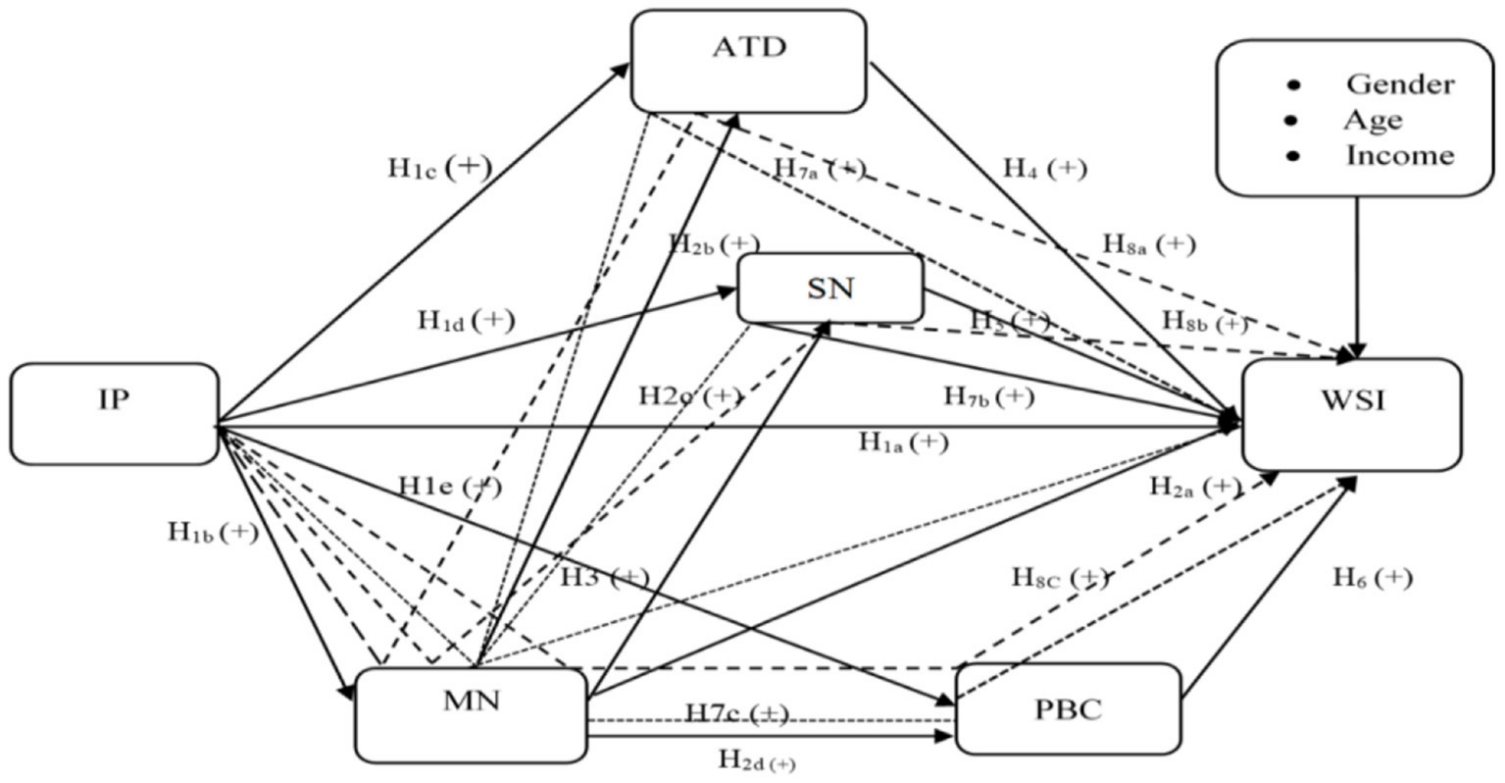
Conceptual framework for waste-sorting intention. IP, information publicity; MN, moral norms; ATD, attitude; SN, subjective norms; PBC, perceived behavioral control; WSI, waste-sorting intention. 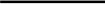 direct effect; ······· mediation; —— serial mediation.

Figure 1 illustrates the sequential mediating effect of moral norms and TPB constructs (ATD, SN, and PBC) in the relationship between IP and WSI.

## Methodology

### Data collection and sampling

This study uses a face-to-face survey technique for the collection of data. Since the present study is focused on WSI by households, therefore purposive sampling technique was employed because it helps in the generalizability of the results where data were collected to ensure a true representation of the population. The main disadvantage of this sampling technique is that bias occurs during the process of sample selection. To minimize bias during data collection, the researcher selected individuals who have an understanding of waste sorting and related concepts. In the past, researchers used purposive sampling techniques to recruit participants who possessed the necessary topic knowledge and understanding of the variables (Khan et al., 2019; Hameed et al., 2022). The questionnaire consists of three parts. The first part was the participants' informed consent stating the study's purpose. This part also ensured the confidentiality of participants' data. The second part was related to demographic information, and the third part collected information on constructs items. We have followed Hair et al.'s (2014) suggested guidelines for calculating the final sample size. The study requires 270 samples (10 responses per item for 27 items). Households were approached in different parts of cities, such as homes, parks, shopping malls, restaurants, and retail stores. Data were collected from 500 individuals from 147 households to enhance our findings' reliability. However, we received many unfilled and multiple response (invalid) questionnaires at the end. The final sample size was 361 after discarding the unfilled and invalid questionnaire. The data collection period ranged from 01 August 2022 to 20 December 2022. Table 1 shows the household demographic information.

**Table 1:** Demographic profile.

		**Frequency**	**Percentage**
Gender	Men	142	30.30
	Women	219	60.70
Age	18–25 years	47	13.01
	26–35 years	156	43.20
	36–45 years	148	41.0
	More than 45 years	10	2.80
Qualification	Intermediate	44	12.20
	Bachelor	153	42.40
	Master/M.Phil	152	42.10
	Ph.D	12	3.30
Household income in PKR	≤ 30,000	150	41.60
	30,001–60,000	62	17.20
	60,001–90,000	33	9.10
	90,001–120,000	39	10.80
	120,001–150,000	29	8.00
	≥150,001	48	13.30

### Instrument

This study adopted measurement scales from past studies to collect household data. Three academic experts from the departments of Management, Marketing, and English have evaluated the content of the final questionnaire. A pilot study involving 45 participants was carried out. The pilot study results were satisfactory as all adopted items of the questionnaire were loaded between 0.60 and 0.70, except one item of information publicity construct that was subsequently modified. After receiving recommendations from experts to make the final questionnaire more understandable for the household, the modifications recommended were incorporated. The experts also reviewed and revised the instruments' items to confirm their content validity. They also identified typos and grammatical errors and suggested layout modifications, which were incorporated into the final questionnaire. **Table 3** shows the number of items adopted and their sources. The measuring items for the attitude and subjective norms scales were adapted from Tonglet et al.'s (2004) study. The measuring items for the perceived behavioral control scale were adapted from Hameed et al.'s (2022) study. The measuring items for the information publicity scale were adapted from Wang et al.'s (2018) study. The measuring items' MN scale was adapted from Botetzagias et al.'s (2015) study. The measuring items for the waste-sorting intention were adapted from the Echegaray and Hansstein (2017) study. A five-point Likert scale was used to measure all items, where one indicated strongly disagree, five indicated strongly agree, and three indicated a neutral response.

The Kaiser–Meyer–Olkin (KMO) test was employed to assess the sampling adequacy of each variable and the overall model. If the significance of Bartlett's test of sphericity is below 0.5, it indicates that the data exhibit strong construct validity. On the contrary, if the KMO value is ≥0.50, it suggests that the sample is sufficient, and higher KMO values indicate greater adequacy (Qalati et al., 2022a). Table 2 shows sample adequacy results. The KMO values exceed 0.7, and Bartlett's test of sphericity sig value is < 0.05. These results indicate that the sample data are sufficient, demonstrating strong construct validity and making it suitable for analysis.

**Table 2:** KMO and Bartlett's test.

Kaiser–Meyer–Olkin measure of sampling adequacy		0.874
Bartlett's test of sphericity	Approx. chi-square	6,038.010
	df	351
	Sig.	0.000

### Common method bias

The presence of common method bias (CMB) poses a risk to the reliability of the gathered data. Therefore, we employed Harman's single-factor test to avoid CMB. The data are not affected by CMB when the amount of variance explained by a single factor is below 50%. The results of Harman's single factor show that the single factor explained a 30.77% variance in the data. Hence, we conclude that CMB is not a threat to the reliability of gathered data (Podsakoff et al., 2003). We also assessed the variance inflation factor (VIF) of all independent variables to ensure that multicollinearity is not a problem. The values of VIF were between 1.390 and 2.926, less than the recommended threshold value of 3.3, depicting no multicollinearity issue (Hair et al., 2021).

## Results

The study utilized two statistical software packages (SPSS) and SmartPLS. SPSS was used for descriptive statistics, common method bias, and multivariate outliers. SmartPLS was used to assess the measurement and structural models.

### Measurement model

The evaluation of the measurement model includes the assessment of reliability and validity. Reliability denotes the consistency and stability of the measuring instrument utilized over time. Validity refers to the extent to which the measuring tool accurately assesses the intended behavior it aims to measure (Yilmaz, 2013). This study uses Cronbach's alpha and composite reliabilities criterion to measure the internal consistency of the data. The values of Cronbach alpha and composite reliabilities above 0.70 indicate internal consistency in the data. Table 3 shows that the study data meet the internal consistency as the values of Cronbach alpha and composite reliabilities were above 0.70. Then, we measured the convergent validity. It shows the degree of constructs relatedness. Convergent validity is confirmed when the average variance extracted (AVE) value is more than 0.50, and factor loading should be at least 0.70 (Hair et al., 2019). The results indicate low factor loadings for the IP3 (0.680) and PBC1 (0.696). Sarstedt et al. (2021) posit that factor loading < 0.70 is common in social science studies. Therefore, we decided not to remove these items. However, the authors suggested the removal of items having low factor loadings (0.40–0.70) from the scale if the deletion of items improves AVE or CR values. First, we followed the same criteria by removing low-loading factors and observed that the removal of a single item resulted in lower factor loadings of the other items such as IP1 and IP2. Thus, following Sarstedt et al. (2021) suggestions, we retained IP3 and PBC1. Table 4 is showing the results of measurement model.

**Table 3:** Constructs' items and sources.

**Constructs**	**Items**	**Item contents**	**References**
Attitude	ATD1	I like the idea of waste sorting.	Tonglet et al., 2004
	ATD2	I have a favorable attitude toward waste sorting.	
	ATD3	Waste sorting makes me feel satisfied.	
Subjective norms	SN1	My friends expect me to manage household waste.	Tonglet et al., 2004
	SN2	My classmates/colleagues expect me to sort waste.	
	SN3	Media influences me when it comes to separating and disposing of waste.	
	SN4	Environmental groups influence me to sort waste.	
	SN5	Most people who hold significance in my life would likely endorse my practice of waste sorting.	
Perceived behavioral control	PBC1	I know what items of waste can be sorted.	Hameed et al., 2022
	PBC2	I know where to take my waste for sorting.	
	PBC3	If I had more information on waste sorting, I believe I would sort more.	
	PBC4	I have plenty of opportunities to sort my household waste.	
	PBC5	The local council provides satisfactory resources for waste sorting.	
Information publicity	IP1	I know that improper handling of waste will pollute the environment and pose a risk to human health.	Wang et al., 2018
	IP2	I am aware that there are many substances present in waste materials.	
	IP3	I think the relevant information publicity for waste sorting is important.	
	IP4	I know the shortcomings of informal waste dumping.	
	IP5	I know what items of household waste can be sorted.	
	IP6	I know where to take my household waste for sorting.	
Moral norms	MN1	It would be wrong of me not to manage household waste.	Botetzagias et al., 2015
	MN2	Not sorting the waste would make me feel guilty.	
	MN3	Not sorting the waste goes against my principles.	
Waste-sorting intention	WSI1	I am willing to get more information about appropriate modes of sorting the waste.	Echegaray and Hansstein, 2017
	WSI2	I intend to manage waste in the next month.	
	WSI3	When I buy home products in future, I tend to choose products that can be easily sorted.	
	WSI4	I intend to participate in the waste-sorting program in the near future.	
	WSI5	I will regularly participate in waste-sorting activities.	

**Table 4:** Reliability testing and convergent validity.

**Constructs**	**Items**	**Loading**	**Cronbach's alpha**	**CR**	**AVE**
Attitude	ATD1	0.898	0.885	0.928	0.812
	ATD2	0.907			
	ATD3	0.899			
Subjective norm	SN1	0.808	0.871	0.912	0.722
	SN2	0.853			
	SN3	0.881			
	SN4	0.854			
Perceived behavioral control	PBC1	0.696	0.826	0.885	0.659
	PBC3	0.832			
	PBC4	0.871			
	PBC5	0.838			
Moral norms	MN1	0.894	0.788	0.874	0.695
	MN2	0.864			
	MN3	0.742			
Information publicity	IP1	0.728	0.822	0.868	0.526
	IP2	0.732			
	IP3	0.680			
	IP4	0.738			
	IP5	0.737			
	IP6	0.735			
Waste-sorting intention	WSI1	0.867	0.921	0.941	0.761
	WSI2	0.822			
	WSI3	0.847			
	WSI4	0.916			
	WSI5	0.907			

### Discriminant validity

Discriminant validity reflects the unrelated relationship among constructs. In other words, it confirms that all variables are unrelated (Hair et al., 2014). We used two criteria to measure the discriminant validity. Fornell and Larcker's (1981) criterion was the first to assess the discriminant validity of the data. According to this criterion, the value of the square of AVEs should be more than the corresponding correlation coefficients. The results of the discriminant validity as per Fornell and Larcker's (1981) criterion are shown in Table 5. The second criterion was the heterotrait–monotrait ratio (HTMT). According to this criterion, the values of the constructs must be < 0.85 to ensure that all constructs are unrelated to each other (Henseler et al., 2009). Table 6 shows that constructs values are < 0.85 confirming that measured constructs are unrelated.

**Table 5:** Fornell and Larcker criterion.

**Latent variables**	**1**	**2**	**3**	**4**	**5**	**6**
Attitude	**0.901**					
Publicity information	0.254	**0.725**				
Moral norms	0.316	0.404	**0.836**			
Perceived behavioral control	0.221	0.095	0.246	**0.812**		
Subjective norms	0.623	0.229	0.320	0.395	**0.849**	
Waste-sorting intention	0.543	0.332	0.413	0.515	0.556	**0.872**

The bold diagonal values refer to the square root of the AVE of each construct. All correlations are statistically significant (p < 0.01).

**Table 6:** Heterotrait–monotrait ratio (HTMT) criterion.

**Latent variables**	**1**	**2**	**3**	**4**	**5**	**6**
Attitude						
Information publicity	0.293					
Moral norms	0.357	0.478				
Perceived behavioral control	0.254	0.200	0.289			
Subjective norms	0.704	0.262	0.367	0.461		
Waste-sorting intention	0.595	0.362	0.459	0.582	0.614	

### Model predictive power

The quality of the model fit depends on its ability to estimate the endogenous construct (Hair et al., 2014). The main criterion for evaluating the inner model is checking the coefficient of determination (R^2^) and cross-validated redundancy (Q^2^; Henseler et al., 2009; Hair et al., 2014). R square (R^2^) evaluates the accuracy of the model prediction. The variance explained by the independent variables on the dependent variable is represented by R^2^ (Hair et al., 2014). Researchers have categorized R square into high, moderate, and low categories. R^2^ is considered strong if its value is more than 0.6, moderate between 0.3 and 0.6, and low below 0.3 (Sanchez, 2013; Khan et al., 2019). The result indicates the value of R^2^ is 54.6% which is near to considerably high. Cross-validated redundancy (Q^2^) is another way to assess the model quality. We have also assessed the predictive relevance of the inner model with (Q^2^) via the blindfolding method. Q^2^ value > 0 confirms the model's predictive relevance (Hair et al., 2014). In this study, the value of Q^2^ is 40.6, which is significantly high, depicting the high quality of the inner model.

### Out of sample power, PLS_predict test

To assess the prediction of the PLS model, we performed out-of-the-sample predictive relevance using PLSpredict (Shmueli et al., 2019). We based our predictive evaluation on root mean squared error (RMSE). Next, we conducted a comparison between the items of the endogenous constructs in PLS-RMSE and the items of the endogenous constructs in the linear model RMSE (LM-RMSE). The results show that most of the PLS-RMSE values are lower than the LM-RMSE values, suggesting that the model exhibits a moderate-to-high level of predictive capability for the endogenous constructs (Hair et al., 2019). In addition, PLS-SEM is preferred over LM due to its higher values of Q^2^_predict for the endogenous items, as shown in Table 7.

**Table 7:** Assessment of PLSpredict power.

**Items**	***Q*^2^_predict**	**PLS-SEM_RMSE**	**LM_RMSE**	**PLS-LM_RMSE**
ATD1	0.007	0.737	0.742	−0.006
ATD2	0.011	0.771	0.775	−0.004
ATD3	0.020	0.698	0.705	−0.006
MN1	0.088	0.860	0.852	0.008
MN2	0.052	0.771	0.757	0.014
MN3	0.087	0.816	0.803	0.012
PBC3	0.031	0.901	0.906	−0.005
PBC4	0.016	1.014	1.020	−0.006
PBC5	0.012	1.002	1.005	−0.002
SN1	0.012	0.774	0.781	−0.006
SN2	0.014	0.921	0.929	−0.008
SN3	0.005	0.805	0.810	−0.005
SN4	0.016	0.813	0.818	−0.005
WSI1	0.066	0.769	0.775	−0.007
WSI2	0.057	0.768	0.774	−0.006
WSI3	0.041	0.757	0.759	−0.003
WSI4	0.081	0.741	0.745	−0.004
WSI5	0.055	0.793	0.797	−0.004

### Hypothesis testing

**Figure 2:**
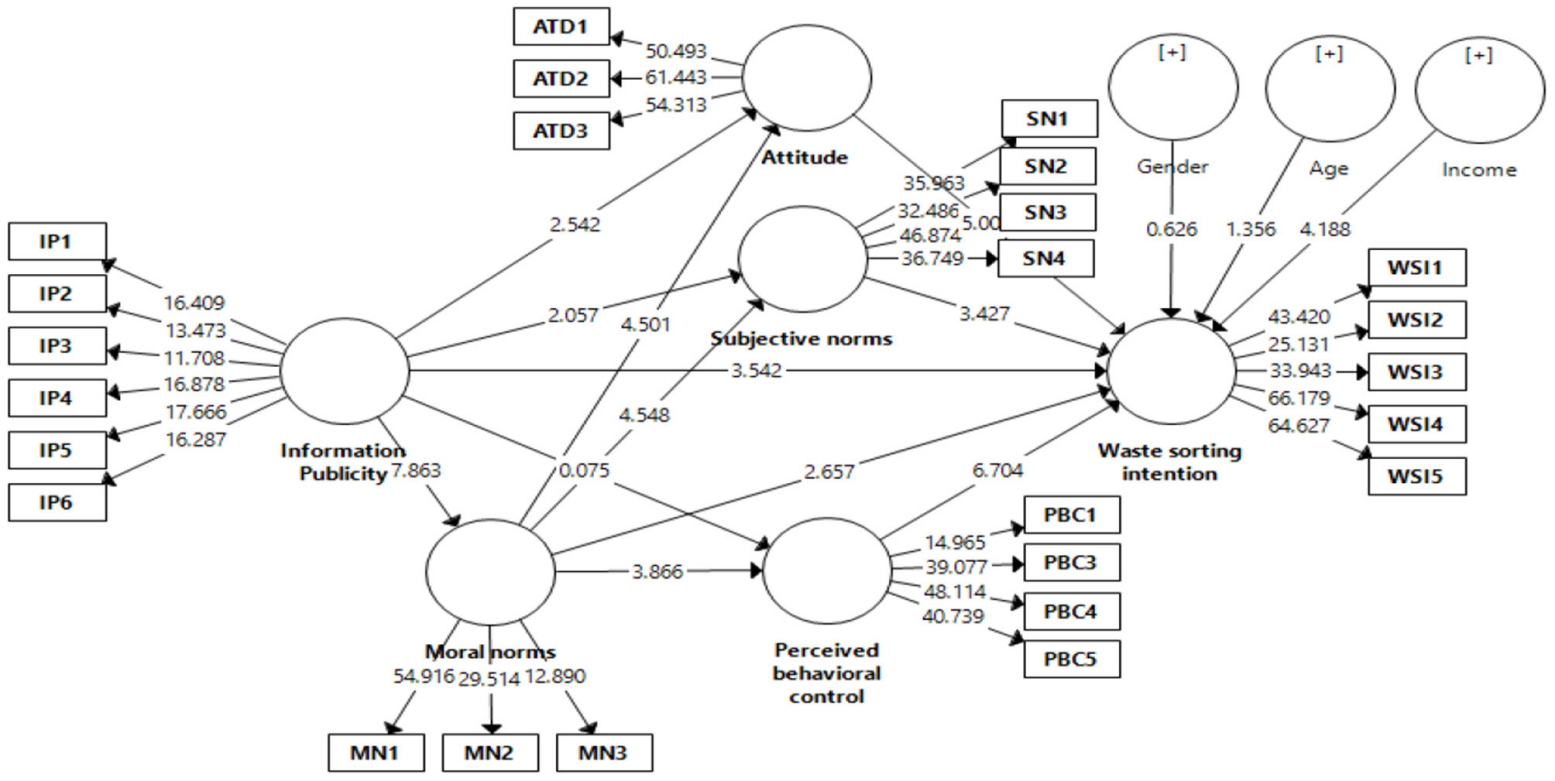
Measurement model.

The study proposed an extended TPB model to measure household waste-sorting intention. To test the hypothesized model, PLS-SEM was performed using the 2000 bootstrapping sampling technique recommended by the researchers (Haenlein and Kaplan, 2004). The direct and indirect effect results are shown in Tables 7, 8, respectively. The results of the direct relationships show that out of 12 hypotheses, only H1e, which proposed the positive influence of IP on PBC, was not supported (β = −0.005; *t* = 0.075; *p* < 0.05). H1a proposed a positive influence of IP on WSI was supported (β = 0.141; *t* = 3.542; *p* > 0.05). H1b proposed a positive influence of IP on MN was supported (β = 0.404; *t* = 7.863; *p* > 0.05). H1c proposed a positive influence of IP on ATD was supported (β = 0.151; *t* = 2.542; *p* > 0.05). H1d proposed a positive influence of IP on SN was supported (β = 0.119; *t* = 2.057; *p* > 0.05). H2a proposed a positive influence of MN on WSI was supported (β = 0.106; *t* = 2.657; *p* > 0.05). H2b proposed a positive influence of MN on ATD was supported (β = 0.255; *t* = 4.501; *p* > 0.05). H2c proposed a positive influence of MN on SN was supported (β = 0.272; *t* = 4.548; *p* > 0.05). H2d proposed a positive influence of MN on PBC was supported (β = 0.248; *t* = 3.866; *p* > 0.05). H4 proposed a positive influence of ATD on WSI was supported (β = 0.277; *t* = 5.005; *p* > 0.05). H5 proposed a positive influence of SN on WSI was supported (β = 0.190; *t* = 3.427; *p* > 0.05). H6 proposed a positive influence of PBC on WSI was supported (β = 0.301; *t* = 6.704; *p* > 0.05). In addition, the study findings revealed a significant impact of income on WSI (β = 1.48; *t* = 4.188; *p* > 0.05). However, the impact of the control variables, age (β = −0.049; *t* = 1.356; *p* < 0.05) and gender (β = 0.022; *t* = 0.626; *p* < 0.05), on WSI was insignificant. Overall, the extended TPB model provides a comprehensive framework for understanding household WSI. Figure 2 is showing the results of structural equation model. These findings show that IP is important in creating awareness and establishing moral norms regarding the proper disposal of waste. With the help of education programs and campaigns, households can learn about waste-sorting benefits such as conserving natural resources, reducing the amount of waste, and preserving the natural environment.

**Table 8:** Hypothesis testing.

**Hypothesis**	**β**	***t*-value**	***p*-value**	**Decision**
**Direct effect**				
H1a: IP → WSI	0.141	3.542	0.001	Supported
H1b: IP → MN	0.404	7.863	0.000	Supported
H1c: IP → ATD	0.151	2.542	0.011	Supported
H1d: IP → SN	0.119	2.057	0.040	Supported
H1e: IP → PBC	−0.005	0.075	0.940	Not supported
H2a: MN → WSI	0.106	2.657	0.008	Supported
H2b: MN → ATD	0.255	4.501	0.000	Supported
H2c: MN → SN	0.272	4.548	0.000	Supported
H2d: MN → PBC	0.248	3.866	0.000	Supported
H4: ATD → WSI	0.277	5.005	0.000	Supported
H5: SN → WSI	0.190	3.427	0.001	Supported
H6: PBC → WSI	0.301	6.704	0.000	Supported
**Indirect effect**				
H3: IP → MN → WSI	0.043	2.390	0.017	Partial mediation
H7a: MN → ATD → WSI	0.070	3.471	0.001	Partial mediation
H7b: MN → SN → WSI	0.052	2.607	0.009	Partial mediation
H7c: MN → PBC → WSI	0.075	3.567	0.000	Partial mediation
H8a: IP → MN → ATD → WSI	0.028	3.148	0.002	Partially sequential mediation
H8b: IP → MN → SN → WSI	0.021	2.390	0.017	No sequential mediation
H8c: IP → MN → PBC → WSI	0.030	2.984	0.003	Partially sequential mediation

All paths are significant; p < 0.05 for two-tailed tests.

### Mediation analysis

The indirect effects were estimated using the bootstrapping technique to examine the mediating role of MN and TPB constructs (ATD, SN, and PBC). We utilized the variance accounted for VAF formula to assess the indirect impact of IP and MN, a mediation analysis approach suggested by Hair et al. (2016). Recently, several studies have used the VAF approach to test mediating effects (Nitzl et al., 2016; Rasoolimanesh et al., 2021; Qalati et al., 2022b). The formula of VAF is given as follows:

VAF = Indirect effect/Total effect

where the total effect is the sum of the direct and indirect effects. According to Hair et al. (2016), VAF values below 20% indicate no mediation, values between 20 and 80% indicate partial mediation, and values above 80% indicate full mediation. The following are the VAF values for the present study:

VAF_H3_: Indirect effect/Total effect = 0.043/0.184 = 23.3%
VAF_H7a_: Indirect effect/Total effect = 0.070/0.176 = 39.7%
VAF_H7b_: Indirect effect/Total effect = 0.052/0.158 = 32.9%
VAF_H7c_: Indirect effect/Total effect = 0.075/0.181 = 41.4%
VAF_H8a_: Indirect effect/Total effect = 0.028/0.139 = 20.1%
VAF_H8b_: Indirect effect/Total effect = 0.021/0.162 = 12.9%
VAF_H8c_: Indirect effect/Total effect = 0.030/0.121 = 24.5%

The results revealed that 23.3, 39.7, 32.9, 41.4, 20.1, and 24.5% indirect effects of H3, H7a, H7b, H7c, H8a, and H8c, respectively, indicate partial mediation, whereas H8b result shows 12.9% indirect effect, indicating no mediation.

## Discussions and conclusion

Waste sorting is one of the best ways to deal with waste, improve environmental health, and protect the planet for future generations. It is the process of separating various forms of waste and arranging them in specified categories for appropriate disposal. Around the globe, people have been encouraged to sort their waste. Different initiatives are used to get households to participate in waste-sorting activities, but in Pakistan, people are not encouraged to sort waste as much as they could. This study used an extended TPB model to predict household waste-sorting intention in Pakistan. The extended TPB discusses how information publicity and MN influence household waste-sorting intentions. This study's findings revealed that the extended TPB model explained 54.6% variance in predicting household waste-sorting intention. The current study suggests that individuals' ATD, SN, and PBC, as well as IP and MN, are essential factors affecting household waste-sorting intention.

The study results revealed that H1a, H1b, H1c, and H1d passed the test related to the positive and significant effects of IP on WSI, MN, ATD, and SN. These findings are consistent with previous studies that argued the importance of IP affecting MN, ATD, and SN (Wang et al., 2018; Waris et al., 2022). The findings indicate that information publicity cannot directly affect the PBC; thus, H1d is rejected, but it does influence indirectly through MN, signifying the importance of IP affecting the MN in the context of waste-sorting intention. The findings are consistent with Wang et al. (2018, 2019), who posit that IP influences MN related to residents' waste separation and recycling behaviors. The results of direct effects revealed that IP has the strongest influence on MN concerning waste-sorting intention. In addition, the findings indicate that ATD, SN, and PBC positively influence WSI. The results support H4, H5, and H6 and are consistent with previous studies where the authors argued that TPB constructs have a crucial impact on the intention to waste separation (Zhang et al., 2015, 2022). It is pertinent to mention that ATD and PBC are the most important predictors of waste-sorting intention. The findings are consistent with Zhang et al. (2015). They found that ATD and PBC were the strongest predictors of residents' waste separation intentions. According to these findings, developing a household's commitment to environmental protection and actively influencing their waste-sorting attitudes are important. PBC increases exert the most influence on household waste-sorting intention. PBC is generally measured by household waste-sorting ability and easiness. Households that have found waste sorting convenient are more likely to engage in waste sorting. If households perceive that waste sorting consumes more time and space, their motivation to waste sorting will reduce. The findings related to mediation and serial mediation indicate that ATD and PBC are the strongest mediators between MN and WSI relationships. Regarding sequential mediating effects, this study's findings revealed that the sequential relationship between IP and WSI is strongest via the inclusion of MN and ATD in the path. This signifies the importance of information publicity influencing household MN and ATD, leading toward WSI. However, the results depict no sequential mediation between IP and WSI via MN and SN sequential path.

## Implications

### Theoretical implications

This study contributes to the TPB theory and enriches the environmental management literature. While earlier research has applied the extended TPB framework to predict waste management, waste separation, and recycling intention (Khan et al., 2019; Hameed et al., 2022; Soomro et al., 2022; Zhang et al., 2022), this study presents an extended TPB framework that simultaneously assesses the impact of IP and MN on waste-sorting intentions. Zhang et al. (2015) and Wang et al. (2018) posited that IP significantly influenced residents' recycling and waste separation intentions. Therefore, they call for exploring relationships between IP and other antecedents of waste management. In addition, prior studies have only assessed the direct and mediating impacts of studied constructs. The current study has proposed a novel framework that assessed the mediating impact of MN. In addition, the current study has proposed serial mediation analysis and enriched the understanding of the relationships among IP, MN, and TPB constructs. The addition of IP and MN is a vital contribution that significantly enhanced the variance in the extended TPB model.

### Practical implications

Practically, this study presents several implications to managers and practitioners who contribute to the betterment of the environment. First, this study reveals that PBC is the most influential factor in shaping waste-sorting intention among households. PBC relates to an individual's ability and tendency to perform a given task. From the study's outcomes, it is clear that households think they can contribute to environmental sustainability through waste-sorting efforts. Therefore, managers and practitioners should focus on the arrangements of campaigns that transform beliefs into behavior and provide helpful information related to waste sorting. The results also depict that MN is an important antecedent of waste-sorting intentions, signifying the importance of household acceptance of responsibility toward protecting the environment. Due to the mismanagement of household waste, the challenge of managing increasing waste is becoming more problematic for the governments of developing nations (Khan et al., 2019; Hameed et al., 2022). Therefore, it is recommended that collective and concentrated efforts be made to promote waste-sorting activities. Researchers argued that people in developing countries have comparatively lower education levels than people in advanced countries (Khan et al., 2019). Therefore, they pay less attention to environmental issues that depict the mismanagement of waste endangering environmental sustainability. Therefore, it is recommended that companies' products for household use should mention the methods of discarding that will eventually lead to environmental protection. The findings indicate the significant impact of income on WSI and show that high-class households are more conscious of waste-sorting activities. Therefore, policymakers should emphasize the middle-class and low-class segment waste-sorting activities to promote environmental sustainability because most of the developing country population consists of middle- and low-class households. To promote waste-sorting activities, the government can incentivize households and encourage them toward waste sorting. The incentive may include rebates on electricity and water sewerage and discounts on garbage collection fees, for households that properly sort their waste. In addition, the government can collaborate with local communities and companies to provide more education and awareness and help create more accessible and convenient ways for households to sort and manage their waste.

## Limitations and future research scope

This study includes some limitations that serve as a guide for future researchers. First, the study's findings are limited to household WSI in Pakistan. Therefore, future studies may better explain the WSI in the context of developing countries by using in-depth interviews and collecting data from diverse groups. Second, the study applied an extended TPB values aspect. Future studies may integrate TPB with the value belief norm and norm activation model to measure the intention comprehensively. Lastly, the study collected data from urban areas having a relatively higher socio-economic profile. Future studies may also consider including data from rural areas comprising more than half the population in developing countries. In addition, researchers may conduct comparative analyses between urban and rural areas to understand people's tendency toward waste sorting.

## Data availability statement

The raw data supporting the conclusions of this article will be made available by the authors, without undue reservation.

## Ethics statement

The studies involving human participants were reviewed and approved by Zhengzhou University Ethics Commitee. The patients/participants provided their written informed consent to participate in this study.

## Author contributions

All authors listed have made a substantial, direct, and intellectual contribution to the work and approved it for publication.
